# Microsatellite development from genome skimming and transcriptome sequencing: comparison of strategies and lessons from frog species

**DOI:** 10.1186/s12864-018-5329-y

**Published:** 2018-12-07

**Authors:** Yun Xia, Wei Luo, Siqi Yuan, Yuchi Zheng, Xiaomao Zeng

**Affiliations:** 1 Chengdu Institute of Biology, Chinese Academy of Sciences, Chengdu, 610041 China; 2 University of Chinese Academy of Sciences, Beijing, 100049 China; 3 College of Bioengineering, Sichuan University of Science & Engineering, Zigong, 643000 China

**Keywords:** Transcriptome, Genome assembly, MiSeq, Amphibians, Population genetics, Next-generation sequencing

## Abstract

**Background:**

Even though microsatellite loci frequently have been isolated using recently developed next-generation sequencing (NGS) techniques, this task is still difficult because of the subsequent polymorphism screening requires a substantial amount of time. Selecting appropriate polymorphic microsatellites is a critical issue for ecological and evolutionary studies. However, the extent to which assembly strategy, read length, sequencing depth, and library layout produce a measurable effect on microsatellite marker development remains unclear. Here, we use six frog species for genome skimming and two frog species for transcriptome sequencing to develop microsatellite markers, and investigate the effect of different isolation strategies on the yield of microsatellites.

**Results:**

The results revealed that the number of isolated microsatellites increases with increased data quantity and read length. Assembly strategy could influence the yield and the polymorphism of microsatellite development. Larger k-mer sizes produced fewer total number of microsatellite loci, but these loci had a longer repeat length, suggesting greater polymorphism. However, the proportion of each type of nucleotide repeats was not affected; dinucleotide repeats were always the dominant type. Finally, the transcriptomic microsatellites displayed lower levels of polymorphisms and were less abundant than genomic microsatellites, but more likely to be functionally linked loci.

**Conclusions:**

These observations provide deep insight into the evolution and distribution of microsatellites and how different isolation strategies affect microsatellite development using NGS.

**Electronic supplementary material:**

The online version of this article (10.1186/s12864-018-5329-y) contains supplementary material, which is available to authorized users.

## Background

Microsatellites, also known as simple sequence repeats (SSRs), are highly polymorphic and generally neutral molecular markers that are still extensively used as genetic markers in evolution and ecology [1–3]. However, the traditional protocols to produce SSRs using microsatellite-enriched libraries, cloning and Sanger sequencing [4] are time consuming and resource intensive. Especially for nonmodel species, microsatellites often have been developed de novo using this process, due to a low rate of cross-species microsatellite amplification success [5, 6]. To overcome these obstacles, next-generation sequencing (NGS) has achieved faster and higher throughput nucleotide sequence data, which facilitate the identification and characterization of microsatellites in nonmodel species. Indeed, hundreds of microsatellite loci were isolated without any screening steps using this high-throughput sequencing technology [7, 8]. Microsatellite loci have been isolated using many NGS platforms, such as Roche 454 [9–11], Ion Torrent PGM [12–14], Illumina [15, 16], and PacBio [17]. The popularity of using NGS not only varies by platforms but also by the sources of the sequences. Both genome skimming and transcriptome sequencing have been used to mine microsatellite markers [18–20].

Although the recent development of NGS techniques has facilitated the de novo development of SSRs, this task is still quite expensive, and the subsequent polymorphism screening requires a substantial amount of time [21, 22]. Obtaining good polymorphic loci usually requires further polymerase chain reaction (PCR) optimization. The mutational dynamics of microsatellites have diversely evolved in different species and in variant repeat types of SSRs [1, 23], and they are important in understanding how to quickly obtain SSRs with high successful amplification rate and non-monomorphic loci for microsatellite isolation. One strategy can efficiently differentiate polymorphic loci from monomorphic loci, by screening multiple genotypes and selecting only those loci that already show differences in repeat length within the set of sequence reads for marker development [21, 24, 25]. Yet, this strategy requires the pooling of multiple genotypes for NGS library construction. Actually, most studies do not make use of such mixed multiple genotypes for library layout [12, 15–17]. Instead, microsatellite data may already or soon be available as a byproduct of NGS. For such cases, we can also use the properties of microsatellites to determine the selection range. A well-known relationship exists between microsatellite length and polymorphism level, due to mutation rate increases with an increasing number of repeat units in an appropriate range [1, 26]. Microsatellites with longer repeat lengths are likely to be highly variable. Intuitively, this seems understandable; more repeat units generally show higher mutation rates because replication slippage may increase in proportion to the number of repeats [1, 27]. In addition, a negative correlation between microsatellite repeat length and substitution rate in nearby flanking sequence was found in mammalian genomes [28]. A low substitution rate in nearby flanking sequences would increase the rate of successful amplification. Getting a high number of repeats may help improve the polymorphism of microsatellites and their allelic richness, especially important for species with low genetic variability.

Researchers often tend to select the most polymorphic markers for subsequent studies and to discard limited polymorphic or monomorphic loci. However, empirical studies have shown that choosing the most polymorphic markers leads to an ascertainment bias and generally overestimates genetic diversity [26, 29]. Furthermore, researchers also tend to select tetranucleotide repeat markers, because they are easier to distinguish, as they cause fewer stutter bands than dinucleotides when screening the polymorphism [1, 23]. However, mutation rates of tetranucleotide loci are varied among species. Some studies reported that tetranucleotide loci have higher mutation rates [13, 30], but not supported in *Drosophila* [31, 32]. Hence, researchers should primarily take this information into account when selecting the appropriate microsatellite loci for ecological and evolutionary studies [29, 33].

The polymorphism of microsatellites is not only affected by the properties of microsatellite repeat units, but also related to the sources of omics data and the assembly of NGS [19, 34]. Genome skimming is by far one of the simplest methodologies for NGS, involving random sampling of a small percentage of total genomic DNA, which can obtain sequences containing plenty of microsatellites [35]. Transcriptome sequencing, or RNA-seq, is also used to isolate SSRs. Microsatellites present in protein coding regions, however, have low allele polymorphism and short repeat lengths, because SSRs could lead to gain or loss of gene function via frame shift mutations [36]. SSR expansions or contractions within genes can eventually lead to phenotypic changes. Yet, microsatellite size homoplasy, characterizing DNA fragments identical in state but not by descent due to convergent evolution, would be more likely occur in genomic SSRs, due to higher mutation rate of genomic SSRs compared to transcriptomic SSRs [37, 38]. Additionally, the assembler chosen for NGS would significantly affect the quality of genome or transcriptome reconstructions, which require special attention during downstream analysis [39, 40]. The microsatellites isolated from NGS could differ depending on sequencing method, read length, library layout, sequencing depth and genome composition. The genome size could also affect the frequency of microsatellites [23, 41]. However, such comprehensive evaluations are only fragmentarily reported in a limited number of species [11, 17]. It remains unclear the extent to which assembly strategy imposes a measurable effect on microsatellite marker development. A deeper understanding of the isolation of microsatellites from NGS is therefore needed, not only to understand evolutionary and mutational properties of microsatellites but also to appropriately use microsatellite markers in ecological and evolutionary studies.

Here, we chose six frog species for genome skimming in two runs of Illumina MiSeq and two frog species for transcriptome sequencing by using Illumina HiSeq to isolate microsatellites for these species. The efficiency of microsatellite development from different sequencing methods and genome composition was compared. Additionally, we investigated how the quantity or sequencing depth of NGS data, read length, and assembly strategy affect the number and polymorphism of the isolated microsatellites.

## Methods

Six frog species were collected for genome skimming by Illumina MiSeq sequencing: *Amolops chunganensis*, *A. mantzorum*, *Hyla chinensis*, *Hylarana latouchii*, *Quasipaa boulengeri*, and *Rhacophorus chenfui*. A single individual for each species was chosen for genomic sequencing. A single individual from two species, *A. mantzorum* and *Q. boulengeri*, were also used for transcriptome sequencing. All the samples were collected in the field of China, and the location and other information for each sample is shown in Additional file 1: Table S1. The frogs in this study were anesthetized and euthanized in 0.2% MS222 (ethyl3-aminobenzoate methanesulfonate) to collect target tissues. Genomic DNA was extracted from muscle tissue using SDS-proteinase K/phenol-chloroform protocol [42]. RNA was extracted individually from six types of tissues (cerebrum, heart, liver, skeletal muscle, skin, and testicle) using TRIzol® reagent according to the manufacturer’s protocol (Life Technologies, Shanghai, China) and mixed with approximately the same quantity. All work with animals was conducted according to relevant national and international guidelines on the Protection of Wildlife (Chengdu Institute of Biology issued permit number CIB#2014–36 and CIB#2014–110). All animal care and experimental procedures were approved by the Animal Care and Use Committee of Chengdu Institute of Biology, Chinese Academy of Sciences (Permit Number: CIB-20121220A). All voucher specimens were deposited in the Herpetological Museum of Chengdu Institute of Biology, Chinese Academy of Sciences.

Genomic DNA and RNA were shipped to Novogene Bioinformatics Technology (Beijing, China) for library construction and sequencing with Illumina MiSeq (PE300) and Illumina HiSeq 2000 (PE125), respectively. Briefly, 3–32 μg of DNA or RNA for each sample is used for library preparation. The concentrations and volumes of DNA and RNA are shown in Additional file 1: Table S1. The paired-end library for genome skimming was prepared using the TruSeq DNA PCR-Free Sample Preparation Kit (Illumina, USA) (insert size approximately 500 bp), following the protocols for Illumina DNA sample preparation. For transcriptome sequencing, libraries were generated using TruSeq SR Cluster Kit v3-cBot-HS (Illumina, USA) following the manufacturer’s recommendations. These libraries were pooled for sequencing on the Illumina platforms (MiSeq for genomic libraries, HiSeq 2000 for transcriptomic libraries) at Novogene Bioinformatics Technology (Beijing, China).

### Contig assembly and microsatellite detection

The raw sequence reads were inspected with FASTQC (http://www.bioinformatics.babraham.ac.uk/projects/fastqc/), and adaptors were trimmed using Trimmomatic [43]. In this step, all raw reads with more than 10% missequenced nucleotides (poly-N) were discarded, and reads with more than 50% of the bases having a Q-value ≤20 were filtered out. De novo assembly of the cleaned reads for genome sequencing for each dataset was performed using SOAPdenovo2 [44] and Trinity [45]. Read preprocessing and assembly parameters followed the individual programs’ guidelines. For SOAPdenovo2, we used 6 k-mer sizes between 55 and 80, with a step size of 5 for the manual specification of assembly parameter values. For each assembly, we monitored the change in total number of contigs and N50 size over the assessed parameter range. For transcriptome sequencing, Trinity and SOAPdenovo2 (8 k-mer sizes between 25 and 60, with a step size of 5) were used for the assembly of the cleaned reads.

The identification of microsatellite loci from the assembly data was performed with QDD3 v3.1.2 [46]. We searched for di-, tri-, tetra-, penta- and hexanucleotide pure repeated units, with at least 7 tandem repeats for dinucleotides, and 6 repeats for the others. To eliminate redundancy, the similarity of sequences containing microsatellite regions was detected by all-against-all BLAST searching. Primer pairs were designed using Primer 3.0 software [47] to meet the following restrictions: the target amplification size range was 120–500 bp, the primer annealing temperature was restricted to 55–65 °C and the primer length was 20–27 bp. In order to avoid the possible bias from software, MISA [48] was also used to identify microsatellite markers, following the default parameter values.

### Length and quantity simulation

We conducted a simulation experiment to investigate the effects that different read length and quantity distributions may have on the discovery of microsatellites and on their primer design. For the read length simulation, we selected the genome sequencing data from *Amolops chunganensis*, and chose five size distributions of reads (100, 125, 150, 175 and 200) for detection. The reduced dataset for each size were performed in ART [49]. The genomic datasets of *Amolops mantzorum* and *Quasipaa boulengeri* were used for data quantity simulation. Five datasets (full, 1/2, 1/4, 1/8, and 1/16 of the total reads) were selected randomly for the quantity simulation. Both the length and the quantity simulation datasets were assembled using SOAPdenovo2 and Trinity. Microsatellite discovery and primer design were performed using QDD3 v3.1.2 and Primers 3.0 software.

### PCR amplification and genotyping

To test the polymorphism and stability of the detected SSRs, we chose *Amolops mantzorum* and *Quasipaa boulengeri* for PCR amplification. The sample information for each species is provided in Additional file 2: Table S2. We synthesized primers for the loci with a target product size of approximately 120–400 bp for amplification. A total of 100 loci for *A. mantzorum*, and 62 loci for *Q. boulengeri* were used for amplification. Eight samples for each species were used for preliminary testing of the primer amplification success rate. Then, electrophoresis was performed with an 8% nondenaturing polyacrylamide gel to evaluate polymorphism. The loci, which could not be successfully amplified, and gave multiple bands, faint bands or bands of unexpected sizes, were discarded from subsequent analyses. Eventually, the forward primers of those loci that showed variation were labeled with one of the fluorescent dyes (FAM, HEX or TAMRA) for a further polymorphism test. For the polymorphism test, we used 64 individuals of *A. mantzorum* and 96 individuals of *Q. boulengeri* to analyses. PCR amplifications were performed in a volume of 15 μl containing 7.5 μl 2× EasyTaq PCR SuperMix (TransGen Biotech, Beijing, China), 0.5 μl each primer (10 pm/μL), 5.5 μl ddH_2_O and approximately 1 μl diluted genomic DNA (50–200 ng/μl). Reaction conditions included an initial 5 min denaturing step at 95 °C, followed by 32 cycles of 95 °C for 30 s, 55–65 °C annealing for 30 s, and 72 °C extension for 30 s, with a final extension of 7 min at 72 °C. The primer sequences, specific annealing temperatures and other information for all the primers used, are shown in Additional file 3: Table S3. The PCR products were run on an ABI-3730xl sequencer and genotyped using GENEMAPPER 4.0 (Applied Biosystems).

The null alleles and genotyping errors for each locus were checked using Microchecker v2.2.3 [50]. The number of alleles, allelic size range, observed and expected heterozygosity were determined using GENEPOP v4.2 [51]. Tests for deviation from Hardy-Weinberg equilibrium (HWE) and linkage disequilibrium (LD) were also calculated using GENEPOP v4.2.

## Results

### Sequence data from Illumina NGS

Genomic sequencing was performed using two runs of Illumina MiSeq, and each run included three frog species (Additional file 1: Table S1). Each run of Illumina MiSeq (PE300) had 22 million raw PE reads. The statistics for sequencing data from each species are shown in Table 1. Each species had almost 4 Gb raw data. For transcriptome sequencing, a total of 125.53 million raw PE reads were obtained from Illumina HiSeq (5.04 Gb and 7.32 Gb, for *Amolops mantzorum* and *Quasipaa boulengeri*, respectively). The raw reads have been deposited in the NCBI SRA database (Bioproject accession number: PRJNA329172, PRJNA347498 and PRJNA374468). After removal of adaptors and low-quality reads, the clean reads were used for the subsequent assembly. The data utilization ratio for Miseq (43.98–85.09%) was much smaller than that for Hiseq (96.27–97.37%) (Additional file 1: Table S1).

**Table 1:** Overview of the sequencing data from Illumina next-generation sequencing for each species of frog

NGS Source	Species	Total sequence reads (Million)	Total No. of bases (Gigabase)
Genomic^a^	*Amolops chunganensis*	7.01	4.21
	*Amolops mantzorum*	10.19	6.11
	*Hyla chinensis*	6.93	4.16
	*Hylarana latouchii*	6.63	3.98
	*Quasipaa boulengeri*	8.28	4.97
	*Rhacophorus chenfui*	7.28	4.37
Transcriptomic^b^	*Amolops mantzorum*	52.28	5.04
	*Quasipaa boulengeri*	73.25	7.32

^a^ Genomic samples were sequenced using the Illumina Miseq (PE300) system

^b^ Transcriptomic samples were sequenced using the Illumina Hiseq 2000 (PE125) system

### NGS assembly and microsatellite detection

**Fig. 1:**
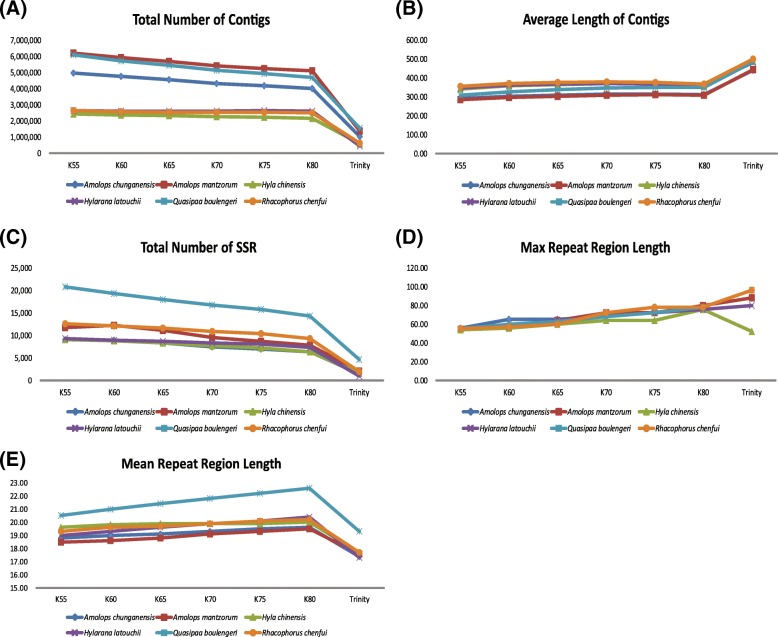
Statistics and assembly results for genome skimming of six frog species using SOAPdenovo2 with different k-mer sizes and Trinity. **a** Total number of contigs; **b** Average length of contigs; **c** Total number of microsatellites isolated using each assembler; **d** Maximum repeat region length of microsatellites for each assembler; **e** Mean repeat region length of microsatellite for each assembler

The total number of contigs and the average length of contigs are shown in Fig. 1. For the SOAPdenovo2 assembly for each species, the smaller k-mer sizes produced more contigs in total, but these contigs had a shorter average length. All the k-mer sizes used for SOAPdenovo2 produced over 2 million contigs for each species, but Trinity produced only a quarter as many contigs. Although fewer contigs were assembled from Trinity than SOAPdenovo2, the Trinity assembler collected contigs with significantly longer average lengths (Fig. 1a, b).

For microsatellite loci detection, each assembly strategy also revealed impressive differences based on QDD results. The smaller k-mer sizes led to the detection of more microsatellite loci with flanking sequences that could be used for primer design (Fig. 1c). Nevertheless, the smaller k-mer size led to the detection of microsatellite loci with lower maximum and mean repeat region length (RRL) (Fig. 1d, e). In contrast, Trinity identified the fewest microsatellite loci and the lowest mean RRL, but with the longest maximum RRL, except in one species (*Hyla chinensis*; Fig. 1). All the assembly strategies produced sufficient numbers of microsatellite loci for further analysis, even the lowest number produced by Trinity was greater than 1000. For the entire dataset, dinucleotide repeats predominate, followed by tri- and tetranucleotide repeats, and penta- and hexanucleotides repeats are least abundant. From the results of MISA, the number of SSRs decreased with increasing k-mer sizes, while the maximum RRL increased with increasing k-mer sizes (Additional file 4: Table S4). Take *Amolops chunganensis* for example, the largest number of SSRs is when K = 60 (196,837), and the smallest number of SSRs is when K = 80 (179885). For the maximum RRL, the smallest is 62 (K = 60), and the largest is 84 (K = 80). The tendency was not affected by the adopted software. Notably, the numbers of identified SSRs were different between the two softwares, since MISA includes all the number of identified SSRs, while QDD only counts the number of identified SSRs with primers.

For RNA-Seq, Trinity is better than SOAPdenovo to obtain average length of contigs and total number of microsatellite loci at any k-mer size (Additional file 5: Table S5). But the maximum and mean RRL in SOAPdenovo were larger than those in Trinity. A total of 121,416 and 278,803 contigs were obtained from Trinity program for *Amolops mantzorum* and *Quasipaa boulengeri*, respectively (Table 2). The total number of contigs for RNA-Seq was much smaller than that obtained for genomic sequencing, but the average length of contigs was longer with RNA-Seq for both species. Both the total number of microsatellite loci and the maximum RRL in RNA-Seq were obviously lower than that obtained from genomic sequencing (*p* < 0.01). Dinucleotide repeats were also the most abundant category, followed by tri- and tetranucleotide repeats, similar to the results from genome sequencing.

**Table 2:** Assembly and microsatellite loci detection statistics for transcriptome sequencing using Trinity

	*Amolops mantzorum*	*Quasipaa boulengeri*
Total number of contigs	121,416	278,803
Average length of contigs	958	919
N50	1953	2080
Total number of SSRs	958	1554
Max RRL^a^	25	32
Mean RRL	16	15.7
Dinucleotide (%)	716 (74.74)	1174 (75.55)
Trinucleotide (%)	226 (23.59)	364 (23.42)
Tetranucleotide (%)	15 (1.57)	14 (0.91)
Pentanucleotide (%)	1 (0.10)	1 (0.06)
Hexanucleotide (%)	0	1 (0.06)

^a^ *RRL* repeat region length, is the length of repeat units

### Data quantity and microsatellite discovery

**Fig. 2:**
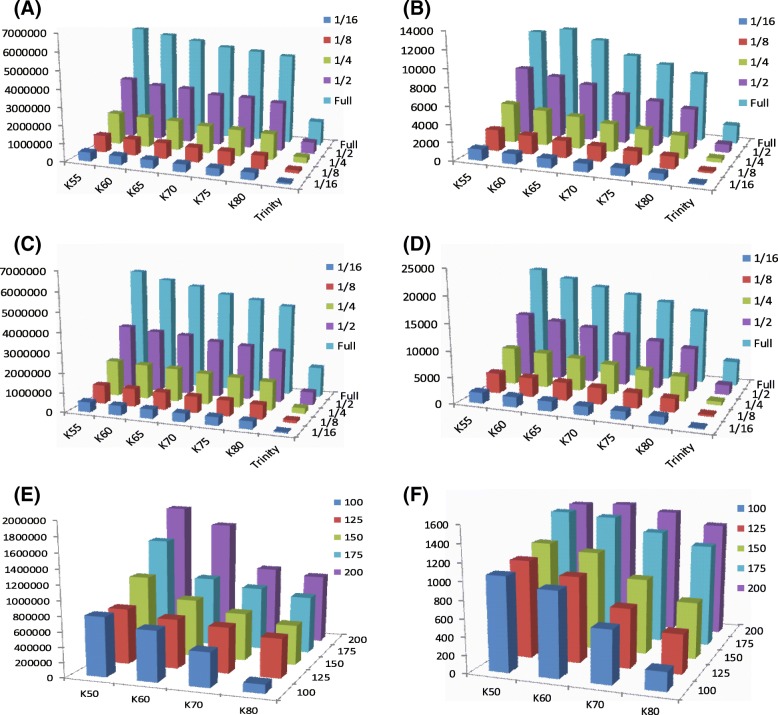
Relation of the k-mer sizes to the data quantity (**a**-**d**) and to the read lengths (**e**, **f**) from genomic datasets. **a** Total number of contigs from data quantity simulation of *Amolops mantzorum*; **b** Total number of identified microsatellites from data quantity simulation of *A. mantzorum*; **c** Total number of contigs from data quantity simulation of *Quasipaa boulengeri*; **d** Total number of identified microsatellites from data quantity simulation of *Q. boulengeri*; **e** Total number of contigs from read length simulation of *A. chunganensis*; **f** Total number of identified microsatellites from read length simulation of *A. chunganensis*

*Amolops mantzorum* and *Quasipaa boulengeri* were chosen for data quantity simulation. The statistics of SSR discovery for each dataset are shown in Table 3 and Fig. 2. The total number of identified microsatellite loci was reduced by the decline in the quantity of data. As expected, the number of microsatellite loci was associated with the size of dataset. For instance, a twofold increase in data quantity, such as from half to full in *Amolops mantzorum*, resulted in a nearly twofold increase in the number of microsatellite loci detected. Furthermore, the number of SSRs for each type of nucleotide repeat also followed this tendency. In other words, the smaller the quantity of NGS data, the fewer the microsatellite loci identified. However, the results showed very little difference in maximum and mean RRL for each dataset (Table 3). The relation of the k-mer sizes to the data quantity reflected that the total number of contigs and microsatellites decreased with increasing k-mer sizes (Fig. 2a-d). There are overlaps of contigs and microsatellites when different k-mers with varied data quantity.

**Table 3:** Effects of data quantity on microsatellite discovery based on genomic data (k-mer size = 65)

	*Amolops mantzorum*					*Quasipaa boulengeri*				
	Full	1/2	1/4	1/8	1/16	Full	1/2	1/4	1/8	1/16
Data in megabases (Mb)	5200	2600	1300	650	327	4700	2400	1200	580	294
Microsatellite loci	11,116	6511	3665	1929	1043	17,942	10,907	6317	3448	1883
Max RRL^a^ (bp)	64	64	60	60	60	66	66	65	65	63
Loci with RRL > 40 bp	241	138	74	36	23	940	561	345	196	118
Mean RRL	18.8	18.8	18.7	18.7	18.6	21.4	21.3	21.3	21.3	21.2
Dinucleotides	8794	5171	2871	1517	836	11,527	7123	4215	2272	1289
Trinucleotides	1640	943	550	300	145	4891	2832	1575	889	447
Tetranucleotides	609	358	219	98	55	1361	854	460	266	140
Pentanucleotides	55	29	18	11	5	127	76	54	18	6
Hexannucleotides	18	10	7	3	2	36	22	13	3	1

^a^ *RRL* repeat region length, is the length of repeat units

### Read length simulation

**Fig. 3:**
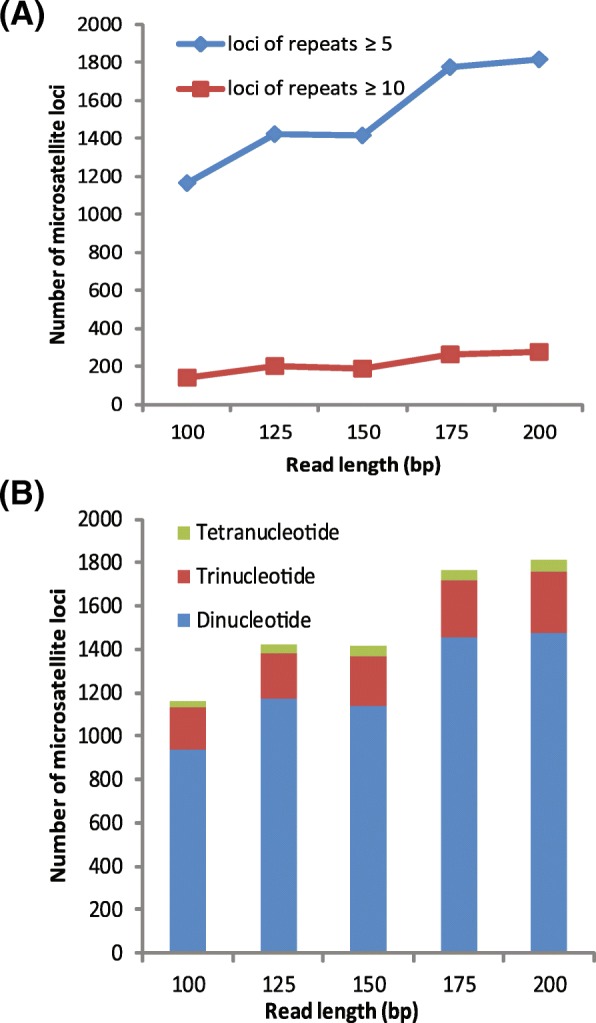
Effects of read length on microsatellite development from genomic datasets of *Amolops chunganensis* (k-mer size = 65). **a** The shorter the read length, the fewer the microsatellite loci identified; **b** The number of dinucleotides, trinucleotides and tetranucleotides identified using different read lengths

To test how the length of the NGS data affects the final quality and the number of SSR loci, we selected the genome sequencing data from *Amolops chunganensis* for length simulation. The results showed a positive relationship between read length and effectiveness of microsatellite isolation (Fig. 3). The shorter the read length, the fewer microsatellite loci were identified. Interestingly, the number of microsatellite loci identified was nearly equal to the read lengths of 125 and 150, implying that for short sequence read assembly, when read length was not increased dramatically, the read length could not directly influence the number of microsatellite loci isolated. The proportion of each type of nucleotide repeat was not affected by read length (Fig. 3b). With increasing k-mer sizes, the total number of contigs and microsatellites would decreases (Fig. 2 e, f).

### Microsatellite development and polymorphism

The characteristics of these newly discovered microsatellites of *Amolops mantzorum* and *Quasipaa boulengeri* are summarized in Additional file 3: Table S3. For *A. mantzorum*, 30 loci could be easily genotyped and displayed polymorphism in 64 individuals from western Sichuan province, China (Additional file 2: Table S2). This population is possibly in Hardy Weinberg equilibrium with loci MT88, MT10, MT84, MT39, MT1, showing signs of a null allele. The number of alleles per locus ranged from 2 to 21 with an average of 9. The observed and expected heterozygosity per locus ranged from 0.078 to 0.984 and from 0.076 to 0.918, respectively (Additional file 3: Table S3). For *Q. boulengeri*, 29 loci showed a clear band of the expected size and displayed polymorphisms in 96 individuals from two populations (Additional file 2: Table S2). The loci QB02, QB18, QB34, QB16, showed signs of a null allele. The number of alleles ranged from 2 to 23, with an average of 7.23. The observed and expected heterozygosity ranged from 0.300 to 0.922 and from 0.237 to 0.937, respectively. A few markers in both species were found to deviate from Hardy-Weinberg equilibrium, suggesting an association with the presence of null alleles (Additional file 2: Table S2).

**Fig. 4:**
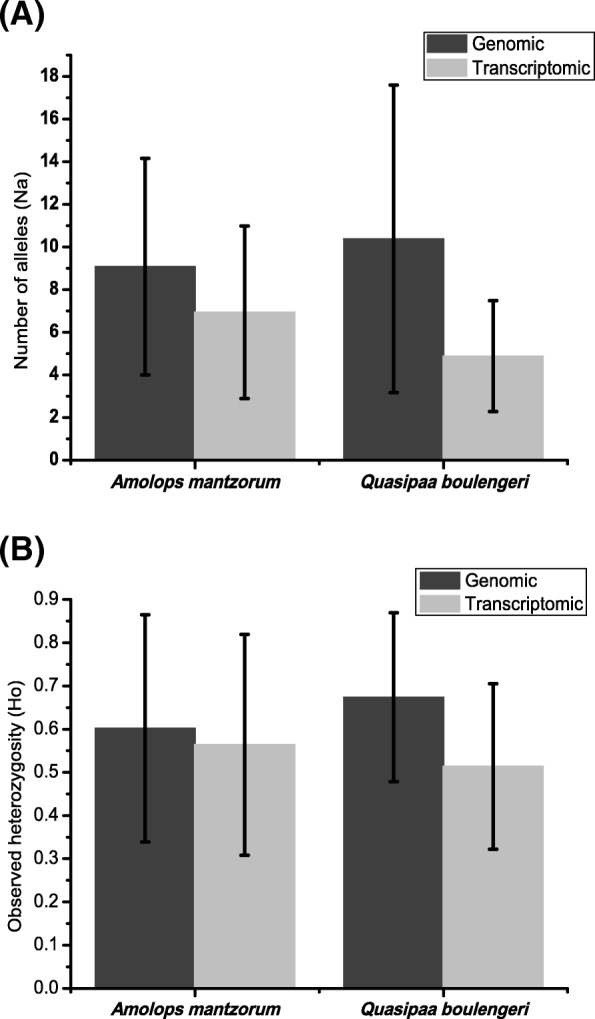
Comparison of the polymorphism between transcriptomic and genomic microsatellite loci within *Amolops mantzorum* and *Quasipaa boulengeri*. **a** The number of alleles (Na); **b** observed heterozygosity (Ho)

**Fig. 5:**
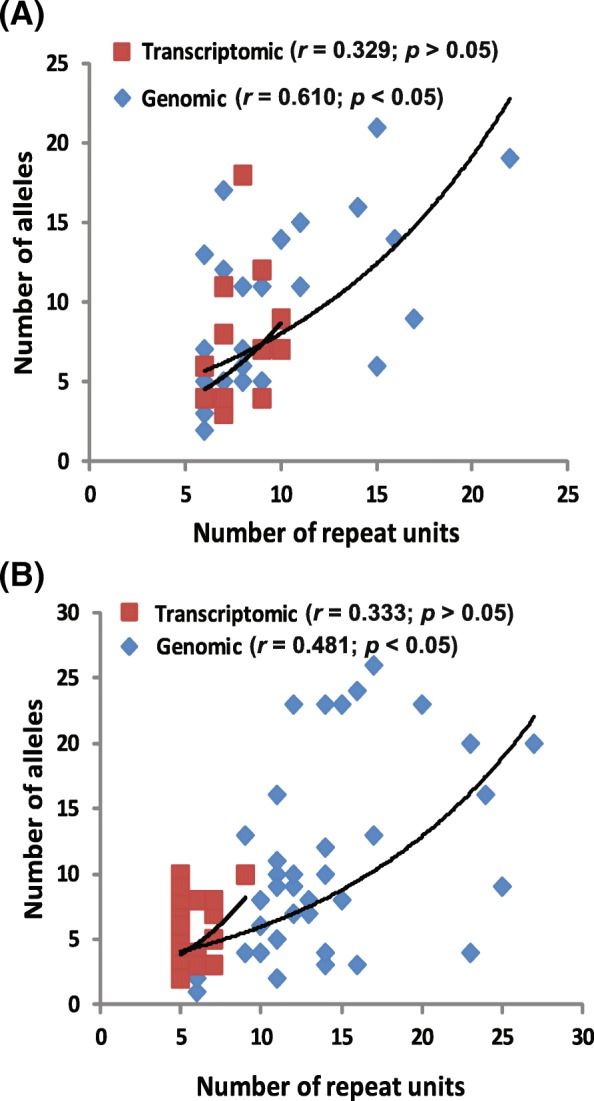
Relation of the number of repeat units to the polymorphism for genomic and transcriptomic microsatellite loci in two frog species. **a** *Amolops mantzorum*; **b** *Quasipaa boulengeri*

Combining the genomic library microsatellites and transcriptome derived microsatellites from previous studies for *Amolops mantzorum* and *Quasipaa boulengeri* [52–54], we compared the properties of microsatellites from different sources. In total, there were 30 genomic and 15 transcriptomic loci for *A. mantzorum*; and 40 genomic and 32 transcriptomic loci for *Q. boulengeri* (Additional file 3: Table S3)*.* Both the number of alleles (Na) and the observed heterozygosity (Ho) were affected by the sources of omics data (Fig. 4). The microsatellites derived from transcriptome have significantly lower Na and Ho values than those from genomic loci in *Quasipaa boulengeri* (independent-samples *T* test, *p* < 0.01), but not significantly lower in *Amolops mantzorum* (*p* > 0.05). In addition, transcriptomic microsatellites possessed fewer repeats and a narrower size distribution than genomic loci (Fig. 5; Additional file 3: Table S3). The number of repeats positively influenced the Na for genomic data (for *A. mantzorum*, Pearson’s correlation coefficient *r* = 0.61, two-tailed *p* < 0.01; for *Q. boulengeri*, Pearson’s correlation coefficient *r* = 0.481, two-tailed *p* < 0.01). However, the number of repeats and Na were not significantly correlated in transcriptomic SSRs (for *A. mantzorum*, Pearson’s correlation coefficient *r* = 0.329, *p* = 0.231; for *Q. boulengeri*, Pearson’s correlation coefficient *r* = 0.333, two-tailed *p* = 0.06).

## Discussion

In the era of NGS, SSRs are still widely used in ecology and evolution [2], because they have their own advantages for population genetics compared to other molecular markers (e.g., single nucleotide polymorphisms, SNPs) [55, 56]. For example, SSRs can be successfully amplified from low quality of DNA (e.g. ancient DNA or museum specimens); the success rate of cross-amplification for SSRs in closely related species is typically higher than that for SNPs [55, 57]. Microsatellites have remained the markers of choice for various population and conservation genetics studies for parentage analysis and for haplotyping in non-model organisms [58, 59]. Specifically, they can provide new insights into genome and chromosome evolution [60, 61]. Their applications in genetics are extensive due to their ceaseless mutation, widespread length variations, co-dominance and reproducibility [1, 23]. Using conventional methods, obtaining nucleotide sequence data for microsatellites and their flanking regions in a target species requires cost and time, particularly for screening, cloning and sequencing microsatellite regions. Utilization of NGS to identify microsatellites is becoming more accessible and feasible, but it still lacks a comprehensive evaluation of how read length, library layout, sequencing depth and assembly strategy influence microsatellite isolation. In this study, we find large variations in the quality of results depending on the different datasets and assemblers used.

### Data quantity, read length, assembly and SSR abundance

Microsatellites are present in the genome of most taxa and are abundant in the genome. Although the abundance of microsatellites differs between taxa and even closely related species, millions of microsatellite loci have been detected in human and mouse genomes [1]. The results from our data quantity simulation revealed that the number of isolated microsatellites increases following an increase in the data quantity when the data quantity is smaller than the entire genome (Table 3, Fig. 2), which can be attributed to the abundance of microsatellites in frog species. In addition, we have isolated these microsatellites randomly or almost randomly from the genome, because there are no dramatic differences in nucleotide repeats and maximum and mean RRL between each dataset. At least two explanations might account for the observation of such a distribution. Firstly, microsatellites may be randomly distributed over the euchromatic genome [62]. However, empirical studies using genome sequencing and fluorescence in situ hybridization (FISH) have shown uneven and nonrandom SSR distribution within and between genomes, where microsatellite density is higher near the centromeres and telomeres [60, 63] . Secondly, the random construction of library layout with genome skimming also resulted in such random isolation. Genome skimming is random sampling of a small percentage of total genomic DNA, which can randomly accept sequences containing microsatellites [35].

The yield of microsatellites through genome skimming could be affected not only by sequencing depth, but also by genome size (C-value). Generally, the frequency of microsatellites correlates positively with genome size [23, 41]. For these six frogs, unfortunately, the genome sizes are unclear, except *Quasipaa boulengeri* [64]*.* The C-value of *Q. boulengeri* is 3.06–3.11 [65, 66]. The coverage for *Q. boulengeri* is approximately 1.6X (4.97 Gigabase). For other species, the coverage could be estimated to 1–2X according to the C-value of the closely related species for these frogs (C-value: 2–7). Due to low coverage sequencing for genome skimming, the data quantity simulation shows the yield of microsatellites for each species increased with increasing sequencing depth (Table 3). However, the highest yield of microsatellites among these species was *Q. boulengeri*, which didn’t have the largest sequencing depth (Fig. 1; Table 1). The number of isolated microsatellites don’t increase following an increase in the data quantity between species. The genome size could affect the frequency of microsatellites. Due to lack of C-value of these species, this assumption need to be further verified. Notably, genome size has a negative effect on amplification success rate [67]. Species with a large genome size, such as salamanders, have a low amplification success rate for microsatellite development [68], due to the relative amounts of target DNA and of available primers for amplifying target DNA could be reduced.

Read length is considered to be one of the most important factors for microsatellite development using NGS [12, 17]. The shorter read lengths can influence SSR development by: 1) reducing the likelihood of discovering longer repeat length microsatellites and 2) reducing the potential success of designing primers [12, 17]. The read length for Illumina is generally shorter than that for the Ion Torrent and the GS-FLX (454) platforms. MiSeq has the longest read lengths (2 × 300 bp) of the Illumina platforms, but its output consists of relatively few reads. HiSeq has shorter reads than MiSeq; the new HiSeq v2 reagents allow 2 × 250 bp in rapid run mode [2]. Despite the shortcoming of read length using Illumina platforms, microsatellite isolation can largely overcome this shortcoming with upgrades in the assembly strategy. After short read assembly, many microsatellite markers have been developed (Fig. 1, Additional file 3: Table S3). Our results (Figs. 2, 3) are in accordance with prior evidence that the shorter the read length, the fewer the microsatellite loci identified [17]. This indicates that microsatellite development from short sequence read assembly would also be influenced by both the length of the flanking regions and the repeat length of microsatellites.

Assembly strategy can also influence the processes involved in microsatellite development. Generally, de Bruijn graph (DBG) based assemblers, such as SOAPdenovo and Trinity, are suited for short read lengths and high-coverage data with less run time and memory complexity [69]. However, the choice of an appropriate k-mer size is one of the major difficulties in using DBG assemblers [39]. Our results show that the quality of both genome reconstructions and microsatellite development depends essentially on k parameter values (Figs.1, 2). The difference of the yields of microsatellites can be attributed to the low coverage of genome skimming and the properties of DBG. When the coverage is low, using a short k-mer for DBG assemblers can be critical for successful assembly, but has a limited ability to resolve repeat sequences, such as microsatellite. In contrast, a longer k-mer size can better resolve repeats but may result in fragmented assembly due to insufficient coverage [70]. Therefore, a longer k-mer size led to the detection of fewer total microsatellite loci and longer repeat length, which have a higher polymorphism level for downstream analysis [26, 27] . For Trinity, because of eliminating both low-complexity and singleton k-mers as initial seeds for contig extensions in Inchworm’s step, it causes the low number of contigs and microsatellites [71]. The longer repeat lengths are not an error of assembly, because they have the same amplification success as shorter repeats (Additional file 2: Table S2, Additional file 3: S3). For transcriptome sequencing, Trinity is better than SOAPdenovo at any k-mer size with regard to average length of contigs and total number of microsatellite loci (Additional file 5: Table S5). It accounts for the high coverage of transcriptome sequencing and algorithmic advantages of Trinity. This observation is consistent with empirical studies that Trinity is suited for de novo transcriptome assembly [72].

### Microsatellite polymorphism

NGS may provide lots of potential microsatellites, but selecting appropriate polymorphic microsatellites is still a critical issue for downstream analysis. Initial polymorphism detection can be performed by pooling multiple individuals when constructing sequencing libraries to investigate whether candidate loci are polymorphic [21, 73]. However, such prior mixing of samples for library construction has not been used in many NGS studies [12, 15–17]. Whatever the methods used, finding polymorphic microsatellite loci is the critical issue. Fortunately, these microsatellites provide repeat type and length, which is indirect information to infer polymorphism. The mutation process seems to be heterogeneous with respect to loci, repeat types and organisms, but it is widely accepted that microsatellites with a greater number of repeats are more mutable [1, 23, 74]. Alleles with a higher number of repeats often mutate at a higher rate, and the relationship between length and rate has been reported to be exponential rather than linear [23]. The results from genomic SSRs revealed that the number of repeats or repeat lengths positively influenced the Na (*p* < 0.01) (Fig. 4), supporting this hypothesis. But these correlations are very weakly for transcriptomic SSRs (*p* > 0.05), and it could be attributed to the functional constraint on transcriptomic SSRs [36]. In addition, those microsatellite loci with long repeat lengths have another advantage in that their nearby flanking sequences have a low substitution rate, which would increase the rate of successful amplification [28]. Of course, a higher mutation rate SSRs with longer repeat length is not necessarily better. Due to ascertainment bias and overestimation of genetic diversity caused by the most polymorphic markers [26, 29], the random selection of SSRs should depend upon the subsequent downstream analyses.

### Transcriptome versus genome microsatellite markers

Both transcriptomic and genomic sequencing identified many potential microsatellites, but the number and properties of these microsatellites were quite different. Transcriptomic SSRs are expected to display lower levels of polymorphism than genomic SSRs, as they are associated with conserved regions of the genome [19, 75]. In our study, the polymorphic screening in *Quasipaa boulengeri* showed that both Na and Ho were lower in transcriptomic than genomic microsatellites (Figs. 4, 5). This low polymorphism could be explained by transcriptomic SSRs are often non-neutral loci, which might bias the estimates of population divergence under the assumption of a neutral model of drift, mutation and migration [19, 34]. In addition, a few study found that the level of SSR variability is likely to differ between coding and untranslated regions [48, 76]. That could be the reason why microsatellites derived from transcriptome were different between *Quasipaa boulengeri* and *Amolops mantzorum*. But it needs to be further investigated. The expansions or contractions of microsatellites within genes can eventually lead to phenotypic changes or loss of function [36]. Though low polymorphism for SSRs from transcriptomic sequencing, some studies would still chose this method to develop functionally linked loci. Because of the functional constraint on transcriptome SSRs, it is more easily to find functional linked loci from transcriptome than genome. The transcriptomic sequence is more conserved than noncoding genome sequence, and more likely to Blast on its sibling species with reference sequences of genome or transcriptome. It is very useful for non-model species to study loci location and function. For *Quasipaa boulengeri*, we employed both genomic and transcriptomic SSRs to isolate sex-linked microsatellite markers. And one locus from transcriptome sequencing data was found [77]. Overall, these results forge a framework for our deep understanding of the evolution and distribution of microsatellites and how different isolation strategies affect microsatellite development using NGS.

## Conclusions

In the era of NGS, microsatellite data may already or soon be available as a byproduct of NGS, and it is still widely used in ecology and evolution. By using genome skimming and transcriptomic sequencing of non-model frog species, we investigated how the assembly strategy, read length, sequencing depth, and library layout affect the number and polymorphism of the isolated microsatellites. These results forge a framework for our deep understanding of the evolution and isolation strategies of microsatellites: 1) the number of isolated microsatellites increases with increased data quantity and read length; 2) assembly strategy could also influence the processes involved in microsatellite development; 3) the transcriptomic microsatellites displayed lower levels of polymorphisms and were less abundant than genomic microsatellites; 4) the proportion of each type of nucleotide repeats was not affected.

## Additional files

Additional file 1: **Table S1.** Summary of sample information for next-generation sequencing and microsatellite discovery. (XLSX 12 kb)

Additional file 2: **Table S2.** Microsatellite screening data of *Amolops mantzorum* and *Quasipaa boulengeri* for each sample. (XLS 94 kb)

Additional file 3: **Table S3.** Characteristics of the microsatellite markers for *Amolops mantzorum* and *Quasipaa boulengeri*. (XLSX 38 kb)

Additional file 4: **Table S4.** Number of identified SSRs by MISA from genomic data of *Amolops chunganensis* and *Amolops mantzorum*. (XLSX 12 kb)

Additional file 5: **Table S5.** Statistics and assembly results for transcriptome sequencing of *Amolops mantzorum* and *Quasipaa boulengeri* using SOAPdenovo2 with different k-mer sizes and Trinity. (XLSX 12 kb)

## Abbreviations

DBG: de Bruijn graph
FISH: Fluorescence in situ hybridization
Ho: observed heterozygosity
Na: Number of alleles
NGS: Next-generation sequencing
PCR: Polymerase chain reaction
RRL: Repeat region length
SNPs: Single nucleotide polymorphisms
SSRs: Simple sequence repeats
